# *Toxoplasma gondii* 70 kDa Heat Shock Protein: Systemic Detection Is Associated with the Death of the Parasites by the Immune Response and Its Increased Expression in the Brain Is Associated with Parasite Replication

**DOI:** 10.1371/journal.pone.0096527

**Published:** 2014-05-06

**Authors:** Paulo Victor Czarnewski Barenco, Elaine Vicente Lourenço, Jair Pereira Cunha-Júnior, Karine Cristine Almeida, Maria Cristina Roque-Barreira, Deise Aparecida Oliveira Silva, Ester Cristina Borges Araújo, Loyane Bertagnolli Coutinho, Mário Cézar Oliveira, Tiago Wilson Patriarca Mineo, José Roberto Mineo, Neide Maria Silva

**Affiliations:** 1 Laboratory of Immunopathology, Institute of Biomedical Sciences, Federal University of Uberlândia, Uberlândia, MG, Brazil; 2 Department of Cellular and Molecular Biology, School of Medicine of Ribeirão Preto, University of São Paulo, Ribeirão Preto, SP, Brazil; 3 Laboratory of Immunotechnology and Immunochemistry, Institute of Biomedical Sciences, Federal University of Uberlândia, Uberlândia, MG, Brazil; 4 Laboratory of Immunoparasitology, Institute of Biomedical Sciences, Federal University of Uberlândia, Uberlândia, MG, Brazil; Pasteur Institute Lille, France

## Abstract

The heat shock protein of *Toxoplasma gondii* (*Tg*HSP70) is a parasite virulence factor that is expressed during *T. gondii* stage conversion. To verify the effect of dexamethasone (DXM)-induced infection reactivation in the *Tg*HSP70-specific humoral immune response and the presence of the protein in the mouse brain, we produced recombinant *Tg*HSP70 and anti-*Tg*HSP70 IgY antibodies to detect the protein, the specific antibody and levels of immune complexes (ICs) systemically, as well as the protein in the brain of resistant (BALB/c) and susceptible (C57BL/6) mice. It was observed higher *Tg*HSP70-specific antibody titers in serum samples of BALB/c compared with C57BL/6 mice. However, the susceptible mice presented the highest levels of *Tg*HSP70 systemically and no detection of specific ICs. The DXM treatment induced increased parasitism and lower inflammatory changes in the brain of C57BL/6, but did not interfere with the cerebral parasitism in BALB/c mice. Additionally, DXM treatment decreased the serological *Tg*HSP70 concentration in both mouse lineages. C57BL/6 mice presented high expression of *Tg*HSP70 in the brain with the progression of infection and under DXM treatment. Taken together, these data indicate that the *Tg*HSP70 release into the bloodstream depends on the death of the parasites mediated by the host immune response, whereas the increased *Tg*HSP70 expression in the brain depends on the multiplication rate of the parasite.

## Introduction

It is estimated that about one-third of the human population worldwide is infected with *T. gondii*. Although toxoplasmosis is asymptomatic in the majority of healthy subjects, different groups of immunocompromised patients might develop severe disease, including those receiving immunosuppressive drugs, organ transplant or HIV-positive patients, in whom the latent reactivation leads to encephalitis [1].

Under stress conditions, *T. gondii* RH strain tachyzoites differentiate into bradyzoites and induce *Tg*HSP70 protein expression [2]. Also, reactivation of toxoplasmosis *in vivo* induces expression of *Tg*HSP70 during bradyzoite to tachyzoite interconversion [3]. *Tg*HSP70 is a highly immunogenic protein [4], which represents a danger signal and a virulence factor in murine toxoplasmosis by modulating the nitric oxide (NO) production by macrophages [5], [6]. Furthermore, recombinant (r*Tg*HSP70) and natural *Tg*HSP70 stimulate the NO release by peritoneal macrophages via TLR2, MyD88 and IRAK4, but under re-stimulation, signaling of r*Tg*HSP70-induced tolerance was mediated by TLR4 [7]. It is well recognized that *Tg*HSP70 activates dendritic cells to produce IL-12 and stimulates spleen B cell proliferation by TLR4-MyD88 pathway [8], [9] as well as auto-antibody formation and IL-10 production by B-1 peritoneal cells [10]. It was also verified that oral *T. gondii* infection with the Fukaya strain induced transient *Tg*HSP70-specific antibodies in BALB/c and C57BL/6 mice, and the higher antibody titers were produced by susceptible C57BL/6 mice [11]. However, the influence of the host genetic background in the *Tg*HSP70 protein expression and the *Tg*HSP70 production systemically or *in situ* as well as the specific humoral immune response under reactivation of infection are widely unknown.

The antigen-specific IgY antibodies have been widely used for research, diagnostic and therapeutic purposes [12]–[14]. Here, we report the use of *Tg*HSP70-specific IgY antibodies as an immunological tool to detect the protein in serum samples of BALB/c and C57BL/6 mice under immunosuppressive treatment with DXM. Also, the effect of the immunosuppression in the protein expression in the brain was evaluated. Our data indicate that the release of *Tg*HSP70 to the bloodstream depends on the death of the parasites mediated by the immune response, as DXM-treated mice presented lower inflammatory alterations, and the higher protein expression in the brain depends on the multiplication rate of *T. gondii*.

## Materials and Methods

### Ethics Statement

All animal experiments were performed in accordance to Brazilian Government's ethical and animal experiment regulations. All experimental procedures were approved by the Animal Experimental Ethics Committee (CEUA) of the Federal University of Uberlândia, under protocol # 060/09. Experiments with chicken immunization were approved by the CEUA under protocol # 107/11. All efforts were made to minimize animal suffering and the numbers of mice required for each experiment.

### Experimental animals

Eight-week old female BALB/c and C57BL/6 mice were purchased from School of Medicine of Ribeirão Preto, University of São Paulo, SP, Brazil and *Calomys callosus* (Rodentia: Cricetidae) were bred and maintained in standard conditions in the Animal Experimentation Laboratory, Institute of Biomedical Sciences, Federal University of Uberlândia, MG, Brazil.

White Leghorn laying hens with 25-weeks age were kindly supplied from Granja Planalto (Uberlândia, MG, Brazil) and maintained in individual cages with water and ration disposal *ad libitum*.

### Parasites and STAg preparation

The ME49 strain of *T. gondii* was used to infect animals in this study. The strain was maintained in *Calomys callosus* that had been inoculated 1 month before with approximately 20 cysts by oral route [15]. The brain cysts of infected animals were collected and used to infect both mice genotypes. Additionally, the serum samples were collected to use in the immunohistochemistry assays as polyclonal antibody to detect the tissue parasitism.

For *T. gondii* soluble antigen (STAg) preparation, tachyzoites of the RH strain were maintained in BALB/c mice by intraperitoneal (i.p.) serial passages at 48-h intervals [16]. The parasites obtained from the peritoneal exudates were washed in phosphate-buffered saline (PBS) and centrifuged at 70×g. The supernatant containing tachyzoites was then pelleted (720×g, 5 min at 4°C), suspended in PBS supplemented with protease inhibitors, sonicated and centrifuged at 10,000×g, 10 min at 4°C, as previously described [17]. The protein concentration was measured by Bradford method [18].

### Recombinant *Tg*HSP70 production and purification

The plasmid pGEx-4T-2 (GE Healthcare, Fairfield, USA) containing *T. gondii* RH *Tg*HSP70 gene (accession number: AF045559.1) in fusion with glutathione-S-transferase gene (GST, 26 kDa) with a cleavage sequence for thrombin was kindly provided by Louis M. Weiss (Albert Einstein College of Medicine, New York, USA) and the recombinant protein (r*Tg*HSP70) was produced as previously described [2]. Isolated colonies were cultured in LB medium (Sigma-Aldrich, St. Louis, USA) supplemented with 100 µg/mL ampicillin and 34 µg/mL chloramphenicol, and incubated at 37°C under agitation until optical density at 600 nm (OD_600_) reached 0.5. Grown cultures were induced with 1 mM isopropyl β-D-1-thiogalactopyranoside (IPTG, Sigma) and incubated at 20°C under agitation for 20 h. Bacterial cells were harvested by centrifugation at 4,000×*g* for 10 min at 4°C. The sediment was resuspended with 3% initial volume of lysis buffer (50 mM Tris-HCl, 150 mM NaCl, 5 mM MgCl_2_, 1 mM EDTA, 1 mM DTT, 1 mM PMSF and 1 mg/mL lyzosime, pH 7.5) (all reagents from Sigma) and incubated at room temperature (RT) for 30 min. After incubation, cells were submitted to freeze-thaw cycles followed by ultrasound disruption on ice and centrifugation at 15,000×*g* for 10 min at 4°C. To obtain the GST-*Tg*HSP70, the supernatant was submitted to affinity chromatography in Glutathione-Sepharose 4 FF column according to manufacturer instructions (GE Healthcare). Briefly, bacterial extract was applied to previously equilibrated column, and after washing, the resin was incubated with 80 U/mL thrombin (Sigma) at RT for 16 h under agitation to obtain pure *Tg*HSP70. Next, the isolated r*Tg*HSP70 protein was submitted to polymixin B affinity chromatography for LPS removal, tested with Limulus Amebocyte Lysate (LAL) (PYROGENT™ Plus, LONZA, Walkersville, USA) and concentrated with Vivaspin 50 MWCO tubes (GE Healthcare). Protein concentration was determined by Bradford assay [18] and samples stored at −20°C until use.

### Chicken immunization and IgY purification

Chicken immunization procedures were performed as previously described [14]. Primary immunization was performed with 100 µg/animal of r*Tg*HSP70 in 250 µL of PBS and equal volume of Freund's complete adjuvant (FCA, Sigma) by intramuscular route. Two boosters were performed at 15-day intervals, with 100 µg of r*Tg*HSP70 plus v/v Freund's incomplete adjuvant (FIA, Sigma) in 500 µL total volume. The individual laid eggs were daily collected and stored at 4°C until further processing. After immunization, eggs laid weekly from each hen were pooled to obtain a weekly kinetics of IgY production.

Egg yolk fat removal and IgY purification were performed as previously described [14]. Egg white was discarded and the yolk was diluted 10 times in deionized water. The pH of solution was adjusted to 5.0 with 0.1 N HCl and then frozen at −20°C for 24 h. After thawing and centrifugation at 10,000×*g* at 4°C for 25 min, lipid-free supernatant was collected and submitted to IgY precipitation with 19% (w/v) sodium sulfate for 2 h. After centrifugation (10,000×*g* at 4°C for 25 min), the pellet was suspended and dialyzed against PBS to eliminate residual salt. Protein concentration was measured by Bradford assay [18] and samples were stored at −20°C until use.

### *C. callosus* immunization and *Tg*HSP70-specific IgG purification

To produce anti-*Tg*HSP70 IgG antibodies, *C. callosus* were injected intraperitoneally with 10 µg/animal r*Tg*HSP70 emulsified in FCA (Sigma). Two more boosters with 10 µg/animal r*Tg*HSP70 emulsified in FIA (Sigma) were performed 15 and 30 days later. After 45 days, serum samples were collected for IgG purification by protein G affinity chromatography according to manufacturer instructions (Sigma). The protein concentration was measured by Bradford assay [18].

### SDS-PAGE and Immunoblotting

Protein samples were analyzed in sodium dodecyl sulfate polyacrylamide gel electrophoresis (SDS-PAGE) in non-reducing condition at 8% for IgY and 10% for *Tg*HSP70 and STAg. Gels were stained with Coomassie blue G-250 (Sigma), documented and analyzed using histogram tool from ImageJ software (NIH, Bethesda, USA). For IgY specificity assay, proteins were transferred to PVDF membranes (Millipore, Billerica, USA) and incubated with 20 mM Tris-buffered saline (TBS) containing 0.1% Tween-20 (TBS-T) and 5% skim milk (TBS-TM) at RT for 2 h. After washing with TBS-T, membranes were incubated with 8 µg/mL anti-*Tg*HSP70 IgY overnight at RT. Membranes were then washed and incubated with 1∶20,000 goat anti-IgY labeled with peroxidase (Sigma) for 2 h at RT. The reaction was revealed with SuperSignal West Pico Chemiluminescent Substrate (Pierce, Rockford, USA) and detected with Amersham Hyperfilm (GE Healthcare). Membranes were documented on a HP Scanjet G4050 scanner (Hewlett-Packard, Palo Alto, USA).

### Indirect ELISA for measuring *Tg*HSP70-specific IgY titers

*Tg*HSP70-specific IgY titration was performed using flat bottom plates (Kartell spa, Noviglio, Italy) coated with 2.5 µg/mL r*Tg*HSP70 or 5 µg/mL STAg diluted in carbonate-bicarbonate buffer (pH 9.6, 50 µL/well) and incubated at 4°C for 16 h. Next, plates were washed with PBS-Tween 0.05% (PBS-T) and incubated with 2 µg/mL of purified IgY samples in triplicate diluted in PBS-T plus 1% skim milk (PBS-TM, 50 µL/well) for 1 h at 37°C. After washing, plates were incubated with Urea 6 M or PBS (50 µL/well) to determine IgY avidity at RT for 10 min. Plates were washed and incubated with peroxidase-labeled rabbit IgG anti-IgY (Sigma) diluted 1∶30,000 in PBS-T (50 µL/well) for 1 h at 37°C. Reaction was developed with o-phenylenediamine (OPD, Sigma) and 0.03% H_2_O_2_. Absorbance was measured at 495 nm in a plate reader (TP Reader, Thermoplate, São Paulo, Brazil). IgY avidity was determined by calculating the ration between the optical density (OD) from urea-treated samples and PBS-treated samples, using the following formula: IgY avidity (%) = OD_urea_/OD_PBS_×100.

### Experimental design and dexamethasone (DXM) treatment

BALB/c and C57BL/6 mice (5 mice per group) were infected i.p. with 10 cysts of ME49 *T. gondii* and sacrificed on days 7, 32 and 56 post-infection (p.i.). In order to evaluate *T. gondii* reactivation, another mouse group (5 mice per group) was treated with 10 mg/mL dexamethasone phosphate in drinking water from 32 to 56 days p.i. [19]. During treatment, mice were observed for weigh change and morbidity scores [20]. Mice were anesthetized (ketamine and xilazine; Syntec, Brazil), the blood was collected and they were sacrificed for tissue sample collection. Brain, lung, liver and spleen tissue samples were processed in two ways: (1) fixed in 10% buffered formalin and embedded in paraffin for histological procedures or (2) immediately frozen at -80°C for further PCR mRNA quantification.

### Histological alterations quantification

Tissue sections were stained with Haematoxilin and Eosin (H&E) for histological assay. Inflammatory scores were analyzed as previously described [21]. Briefly, perivascular cuffs and inflammatory cells in the meninges as well as total focal or diffuse inflammatory foci were analyzed in a sagittal section. The inflammatory score was represented as arbitrary units: 0–2, mild; 2–4, moderate; 4–6, severe; and above 6, very severe. The histological analyses were done in two histological sections from each mouse using a 40× objective by two researchers in a blind manner.

### Immunohistochemistry assays for parasite burden and *Tg*HSP70 quantification

The tissue parasitism was evaluated in the organs by immunohistochemistry as previously described [21]. To detect parasite antigen, deparaffinized sections were incubated at RT with PBS plus 3% nonfat milk to reduce non-specific binding and then incubated at 4°C overnight with *C. callosus* anti-*T. gondii* polyclonal antibodies, produced by infecting *C. callosus* with ME-49 strain of *T. gondii*, diluted in 0.01% saponin. After incubation with 1∶300 biotinylated goat anti-mouse antibodies (Sigma) that recognizes *C. callosus* immunoglobulin, the assay sensitivity was improved by avidin-biotin-peroxidase complex (ABC kit, PK-4000; Vector Laboratories, Inc., Burlingame, USA). The reaction was visualized by incubating the section with 3,3′-diaminobenzidine tetrahydrochloride (DAB, Sigma) for 5 min. Control slides were incubated with serum of non-infected *C. callosus*. The slides were counterstaining with Harris Haematoxylin and analyzed with an Olympus microscope.

The tissue parasitism detected by immunohistochemistry was scored by counting the number of parasitophorous vacuoles and cyst like structures from forty microscopic fields in the lung, liver and spleen or per section in the brain of mice infected with *T. gondii* using a 40 x objective. Two noncontiguous histological sections of each mouse (40 µm distance between sections) from five mice per group were examined.

Photomicrographs of tissue section obtained using a 20× objective (HLImage++, Western Vision Software, Salt Lake City, USA) were analyzed by the Microsoft Image Composite Editor (Microsoft, Redmont, USA) to create whole tissue panorama images. Areas in those images were quantified using threshold and measure tools from ImageJ software. Parasite burden was determined by calculating the ratio between the parasite number and the respective slice area.

To detect *Tg*HSP70 in tissue sections, 30 µg/mL of purified anti-*Tg*HSP70 IgY and peroxidase-labeled rabbit anti-chicken IgY (Sigma) diluted 1∶500 were used as primary and secondary antibodies, respectively, and the reaction was revealed with DAB (Sigma), as described above. For *Tg*HSP70 quantification, photomicrographs of tissue section were obtained using a 40× objective (LAS EZ, Leica, Wetzlar, Germany) from each cerebral cyst. The brown (DAB) staining intensity was quantified using color deconvolution plugin [22] in ImageJ, which differentiates brown staining from tissue Haematoxilin-blue background color. Cyst-staining patterns were determined using ImageJ histogram tool that counts frequencies (F_D_) for each DAB-brown intensity scale. The DAB-brown intensity (I_D_) color binary scale was linearly converted to 0-to-1 scale. *Tg*HSP70 intensity was calculated by the sum of each DAB-brown intensity multiplied by its respective frequency, as follows:
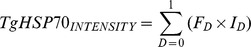



### ELISAs to detect STAg- and *Tg*HSP70-specific IgG antibodies, *Tg*HSP70, and *Tg*HSP70-antibody immune complexes in serum samples

Levels of STAg- and *Tg*HSP70-specific IgG antibodies were measured by ELISA as described elsewhere [23], with modifications. Microtiter plates (Kartell) were coated with 5 µg/mL STAg or 10 µg/mL *Tg*HSP70 diluted in carbonate-bicarbonate buffer (pH 9.6, 50 µL/well) at 4°C for 16 h. After washing, mouse sera diluted 1∶25 (*Tg*HSP70) and 1∶64 (STAg) in PBS-Tween (PBS-T) plus 1% skim milk were added in triplicate to plates (50 µL/well). After incubation at 37°C for 1 h, plates were washed and incubated with peroxidase-labeled goat anti-mouse IgG (Sigma) (50 µL/well) diluted 1∶1,000 at 37°C for 1 h. Reaction was developed with enzyme substrate consisting of 0.03% H_2_O_2_ and 0.5 mg/mL OPD (Sigma) (50 µL/well). The optical density (OD) was measured at 495 nm by using a plate reader (TP Reader, Thermoplate). Specific IgG1 and IgG2a assays were performed similarly using mouse serum diluted 1∶10 for both reactions and detection antibodies diluted 1∶6,000 and 1∶2,000 (Sigma), respectively. As control, plates were incubated with sera of non-infected mice.

Circulating *Tg*HSP70 quantification assay was carried out using high-binding microtiter plates (Costar-Corning, Tewksbury, USA) coated with 10 µg/mL of anti-*Tg*HSP70 *C. callosus* IgG (produced as described above) diluted in carbonate-bicarbonate buffer at 4°C for 16 h. After washing, plates were blocked with PBS-T plus 5% skim milk for 1 h and then incubated with mouse sera diluted 1∶40 at 37°C for 1 h. Next, plates were washed and incubated with 25 µg/mL of purified anti-*Tg*HSP70 IgY. Also, a standard curve with 11 concentrations of serially two-fold diluted r*Tg*HSP70 (5 µg/mL to 4.9 ng/mL) was added to each plate. After incubation at 37°C for 1 h, the peroxidase-labeled rabbit anti-chicken IgY (Sigma) diluted 1∶30,000 was added to plates and incubated at 37°C for 1 h. Reactions were developed with OPD and the absorbance was measured as described above. The concentration of *Tg*HSP70 in serum samples was determined by comparison with the standard curve. Circulating ICs titration assay was performed as described previously [24] with modifications. Microtiter plates (Kartell) were coated with 10 µg/mL anti-*Tg*HSP70 IgY diluted in carbonate-bicarbonate buffer at 4°C for 16 h. After washing, plates were incubated with mouse sera diluted 1∶40 at 37°C for 1 h. Next, plates were washed and incubated with peroxidase-labeled goat anti-mouse IgG (Sigma) diluted 1∶1,000 at 37°C for 1 h. Reactions were developed and analyzed as described above.

All serological results were expressed as ELISA index (EI) as follows: EI = OD_sample_/cut-off, where cut-off was established as mean OD values of negative control sera plus three standard deviations. Based on screening tests performed with negative and positive control sera, EI>1.2 values were considered positive results.

### Quantitative PCR for mRNA expression

Brain sample mRNA was harvested using TRIzol reagent according to manufacturer instructions (Life Technologies, Carlsbad, USA). RNA concentration was determined (GeneQuant 1300 spectrophotometer, GE Healthcare) and complementary DNA (cDNA) was synthesized using 150 ng mRNA through reverse transcription reaction following manufacturer instructions (Promega, Madison, USA). Quantitative PCR (qPCR) assays were performed using SYBR green reagent (Roche, Basel, Switzerland) in Applied Biosystems 7500 Real-Time PCR System (Life Technologies). Assays were performed at 95°C for 10 min and 40 cycles at 94°C (1 min), 62°C (2 min) and 72°C (1 min). Specific primers used for murine GAPDH, IFN-γ, TNF, IL-10, IL-1β and for *T. gondii* antigens, tachyzoite (SAG1), and *Tg*HSP70 from RH strain and bradyzoite (BAG1) from ME49 strain mRNA quantification were designed using Primer Express software (Life Technologies) based on GenBank sequences (Table 1). BLAST alignments of *Toxoplasma* mRNA nucleotides indicate that our primer sets is equally highly specific for most *T. gondii* strains, including ME49. Relative quantification of *T. gondii* and murine cytokines mRNA to murine GAPDH was performed using the 2^-ΔΔCt^ method [25].

**Table 1:** List of primer sequences used for *T. gondii* qPCR gene expression assays.

Gene name	GeneBank Accession No.	Primers sequences
*m*GAPDH	NM_008084.2	FW: 5′-GGAGAAACCTGCCAAGTATGATG-3′
		RV: 5′-CAGTGTAGCCCAAGATGCCC-3′
*m*IFN-γ	NM_008337.3	FW: 5′- GGCTGTTTCTGGCTGTTACTGC-3′
		RV: 5′- CATCCTTTTGCCAGTTCCTCC-3′
*m*TNF	NM_013693.2	FW: 5′-CCACCACGCTCTTCTGTCTACTG-3′
		RV: 5′- GATCTGAGTGTGAGGGTCTGGG-3′
*m*IL-1β	NM_008361.3	FW: 5′- CCAAAAGATGAAGGGCTGCT-3′
		RV: 5′- GCTCTTGTTGATGTGCTGCTG-3′
*m*IL-10	NM_010548.2	FW: 5′- TTTAAGGGTTACTTGGGTTGCC-3′
		RV: 5′- CGCATCCTGAGGGTCTTCA-3′
*Tg*BAG1	NW_002234536.1	FW: 5′- GGAGCCATCGTTATCAAAGGAG-3′
		RV: 5′- TTTGCCATCGTCCACTTTCTC-3′
*Tg*HSP70	AF045559.1	FW: 5′- TCGGCAAGGAAGTGAAGGAG-3′
		RV: 5′- TTGATAATGCGGAGGACGCT-3′
*Tg*SAG1	AY661791.1	FW: 5′-TTTCCGAAGGCAGTGAGACG-3′
		RV: 5′-CCATAACGCCACATCGCA-3′

Italic letters before gene names represent: *m* = *Mus musculus*; *Tg* = *T. gondii*.

### Statistical analysis

Statistical analyses were performed using GraphPad Prism 5 (GraphPad Software, San Diego, USA). Data were expressed as mean ± SEM. Data from groups within the same mouse lineage were compared using one-way ANOVA and Bonferroni post-test (symbol  =  *; bar graphs), and comparison between lineages of the same group were analyzed with Student's *t* test (symbol = †). Correlation between variables were tested with Pearson's test (symbol = *; plot graphs). Differences were considered significant when *P*<0.05.

## Results

### Immunized chickens produce high-avidity *Tg*HSP70-specific IgY

**Figure 1:**
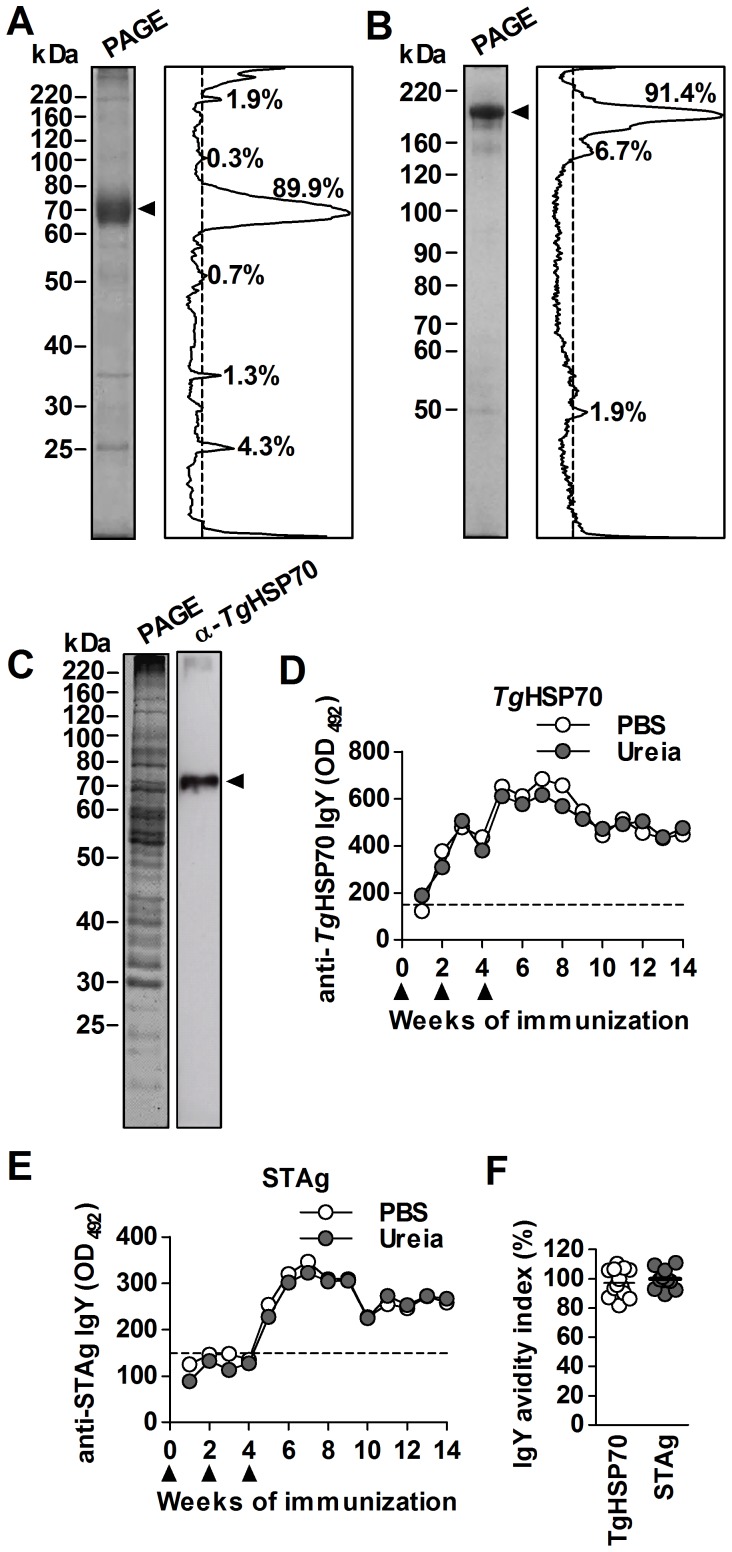
Production of high-avidity *Tg*HSP70-specific IgY antibodies. (**A**) The r*Tg*HSP70 protein in fusion with GST was expressed in *E. coli* and purified by glutathione and polimixin B affinity chromatography. Purified fraction was submitted to 10% SDS-PAGE (left) and grey-scale band profile was analyzed with ImageJ software (right), indicating a 70 kDa protein with 89.9% of purity (arrow head). (**B**) ACF-emulsified r*Tg*HSP70 was used to immunize Leghorn chickens and produce *Tg*HSP70-specific IgY antibodies. Na_2_SO_4_-precipitated egg yolk antibody was subjected to 8% SDS-PAGE (left) and grey-scale band profile was analyzed with ImageJ software (right), showing a 180 kDa protein with 91.4% of purity (arrow head). **C**: STAg proteins were separated with 10% SDS-PAGE (left) and transferred to PVDF membrane, which was incubated with anti-*Tg*HSP70 IgY antibody (right). A unique 70 kDa band was observed (arrow head). (**D** and **E**) Kinetics of anti-*Tg*HSP70 IgY production after immunization of chickens. Samples were analyzed in the presence (urea) or absence (PBS) of urea 6 M to evaluate IgY avidity to *Tg*HSP70, using r*Tg*HSP70 (**D**) or STAg (**E**) as antigen. Avidity index (%) of anti-*Tg*HSP70 IgY for both r*Tg*HSP70 and STAg antigens (**F**). Avidity index was calculated as the ratio between Urea OD_492_ and PBS OD_492_ values X 100. Black arrow heads in **D** and **E** indicate the weeks in which chicken were immunized. Gels from **A** to **C** were stained with Coomassie blue G-250. kDa, kiloDaltons.

The r*Tg*HSP70 protein was obtained at high purity degree as shown by the SDS-PAGE electrophoretic profile and grey-scale band profile analyzed by ImageJ software, indicating a 70 kDa protein with 90% of purity (Figure 1A). Next, ACF-emulsified r*Tg*HSP70 was used to immunize chickens in order to obtain specific IgY antibodies. Daily collected eggs from r*Tg*HSP70-immunized hens contained about 15 mL of yolk content, in which protein concentration was 3.0±0.16 mg/mL, totalizing 45 mg per egg. Na_2_SO_4_-precipitated egg yolk samples consisted of IgY fractions migrating at 180 kDa in 8% SDS-PAGE with 91% of purity when analyzed by grey-scale band profile (Figure 1B). Immunoblotting assays were performed to determine IgY specificity to r*Tg*HSP70. For this purpose, STAg proteins were run in 10% SDS-PAGE and blotted on PVDF membrane, which was incubated with anti-*Tg*HSP70 IgY purified fraction. Only a 70 kDa band was detected (Figure 1C). The kinetics of anti-*Tg*HSP70 IgY production was weekly analyzed by ELISA during the immunization period. Anti-*Tg*HSP70 IgY antibodies were detected from the 2^nd^ week and forth, with a maximum reactivity on the 7^th^ week of immunization and the antibody production persisted until 14 weeks post-immunization (Figure 1 D). Only after the 4^th^ week of immunization anti-*Tg*HSP70 IgY antibodies were able to detect natural *Tg*HSP70 protein in the STAg extract, with maximum reactivity at 7^th^ week post-immunization (Figure 1 E). Additionally, both assays were performed in the presence of urea, to determine *Tg*HSP70-specific IgY avidity and the results showed reactivity similar to non-treated samples (PBS) (Figure 1, D and E). Thus, *Tg*HSP70-specific IgY antibodies obtained from egg yolk of immunized chickens presented high avidity profile, since the ratio of urea-treated samples per non-treated samples was nearly 100% on average (Figure 1F). These data indicate that immunized chickens produce IgY antibodies against *Tg*HSP70 in the egg yolk with high avidity.

### DXM treatment decreased inflammation and increased parasite load in C57BL/6 mice

**Figure 2:**
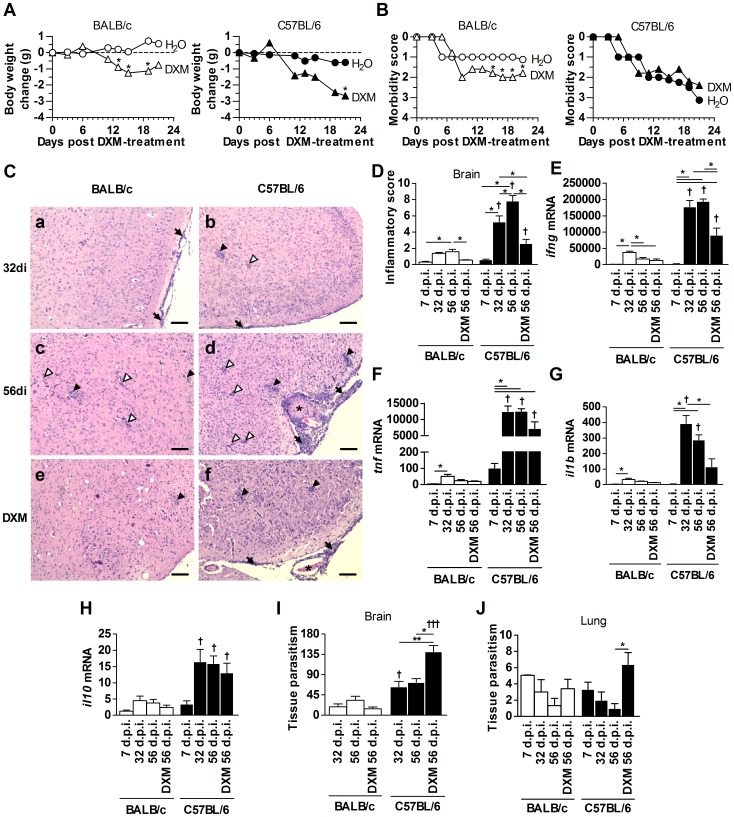
DXM treatment decreased inflammatory alterations and increased parasite load in chronically infected C57BL/6 mice. BALB/c and C57BL/6 mice were treated with DXM during 24 days, beginning on day 32 p.i. and kinetics of inflammatory changes and parasite burden were analyzed. Both mouse lineages were observed for body weight change (**A**) and morbidity score (**B**) during DXM treatment. Illustrative photomicrographs of brain tissue sections stained with H&E (**C**) from BALB/c and C57BL/6 mice infected for 32 days (**a** and **b**), 56 days (**c** and **d**) and from mice DXM-treated and infected (**e** and **f**). Bar scale, 100 µm. The black arrows indicate the inflammatory cell infiltrates in the meninges; black arrowheads indicate the inflammatory cell infiltrates in the parenchyma; white arrow heads indicate vascular cuffings; and asterisk indicates thrombus. The data of inflammatory score in the brain (**D**) were obtained by analyzing 40 microscopic fields per section, on six sections from each mouse using a 40× objective. The cytokine IFN-γ (**E**), TNF (**F**), IL-1β (**G**) and IL-10 (**H**) mRNA expression in the brain were analyzed by qPCR. The quantification of tissue parasitism (cyst-like structures and parasitophorous vacuoles) by *T. gondii* in the brain (**I**) and lung (**J**) of infected mice were done by immunohistochemistry assays. Data are representative of at least two independent experiments of 5 mice per group that provided similar results. *Significant differences between different treatment conditions within the same mouse lineage (one-way ANOVA and Bonferroni multiple comparison post-test; *P<0.05). ^†^Significant differences between the two mouse lineages submitted to the same treatment conditions (Student's *t* test; ^†^ *P*<0.05).

BALB/c and C57BL/6 mice were infected with 10 cysts of the *T. gondii* ME49 strain by intraperitoneal route and treated with DXM after 32 days p.i. to induce immunosuppression. During 24 days of DXM-treatment, mice were examined for weight loss and morbidity score. It was observed that DXM-treated and infected BALB/c mice presented higher body weight loss compared with infected and untreated animals of the same lineage; however, DXM-treated and infected C57BL/6 mice presented progressive and more severe weight loss compared with infected animals (Figure 2A). Additionally, DXM-treated BALB/c and C57BL/6 mice treated or not with DXM presented high morbidity score, such as ruffled coat followed by demeanor symptoms, such as tottering gait and reluctance to move (Figure 2B). The symptoms were more severe and progressive in C57BL/6 DXM-treated or not and infected mice (Figure 2B). In order to observe the effects of DXM in encephalitis, brain tissue sections were stained with H&E and analyzed for histological alterations. On day 7 p.i., mice of both lineages presented small number of inflammatory foci. On day 32 p.i. C57BL/6 mice presented lesions in the brain that were characterized by mononucleated cell infiltrates in the parenchyma, glial nodules, vascular cuffing by lymphocytes and focal mononucleated cell infiltrates in the meninges, and the lesions were more severe in this mouse lineage compared with BALB/c mice (Figure 2C, panels **a** and **b**). On day 56 p.i. BALB/c mice presented higher inflammatory score in the organ compared with mice of the same lineage on day 7 p.i. and in comparison with DXM-treated BALB/c mice (Figure 2C, panels **c** and **e**, and 2D). C57BL/6 mice presented higher inflammatory score in the brain on day 56 p.i. compared with those on days 7, 32, and 56 p.i. with DXM-treatment (Figure 2C, panels **b**, **d** and **f**, and 2D). Additionally, C57BL/6 mice presented higher inflammatory score in the brain on days 32, 56 and DXM-treated mice on day 56 p.i. compared with BALB/c mice in the same condition (Figure 2D). In parallel, the transcripts of the proinflammatory, IFN-γ, TNF, IL-1β and regulatory, IL-10, cytokines were measured in the brain of infected mice. In accordance with higher inflammation in the brain, C57BL/6 mice presented higher IFN-γ and TNF mRNA expression levels compared with BALB/c mice on days 32, 56 and DXM-treated mice on day 56 p.i. (Figure 2E and F), and higher IL-1β on days 32 and 56 p.i. (Figure 2G). Interestingly, C57BL/6 mice also presented higher IL-10 mRNA expression levels compared with BALB/c mice on days 32, 56 and DXM-treated mice on day 56 p.i. (Figure 2H).

The parasite load was also analyzed in the brain tissue sections of DXM-treated or not and infected mice. C57BL/6 on day 32 p.i. and 56 p.i. DXM-treated mice presented higher number of parasitophorous vacuoles and cyst like structures in the brain compared with BALB/c mice in the same condition (Figure 2I and Figure S1 A, C and D). Moreover, DXM-treatment promoted opposite effects in BALB/c and C57BL/6 mice. DXM-treated BALB/c mice presented lower parasite load whereas DXM-treated C57BL/6 mice presented higher *T. gondii* parasitism in the brain on day 56 p.i. (Figure 2I and Figure S1 C and D).

The parasite load was also analyzed in the peripheral organs of DXM-treated or not and infected mice. Parasites were seldom found in the spleen and in the lungs of BALB/c and C57BL/6 mice on day 56 p.i. and in those treated with DXM. DXM-treatment induced an increase in the parasite count in the lungs of C57BL/6 mice compared with non-treated mice in the same period of infection, although the total number of parasites remains low in both lineages (Figure 2J and Figure S1 B). No parasite was found in the most of spleen and liver samples (data not shown).

### *T. gondii* infection induces anti-*Tg*HSP70 IgG production and circulating *Tg*HSP70 in BALB/c and C57BL/6 mice, but specific immune complexes occur only in resistant mice

**Figure 3:**
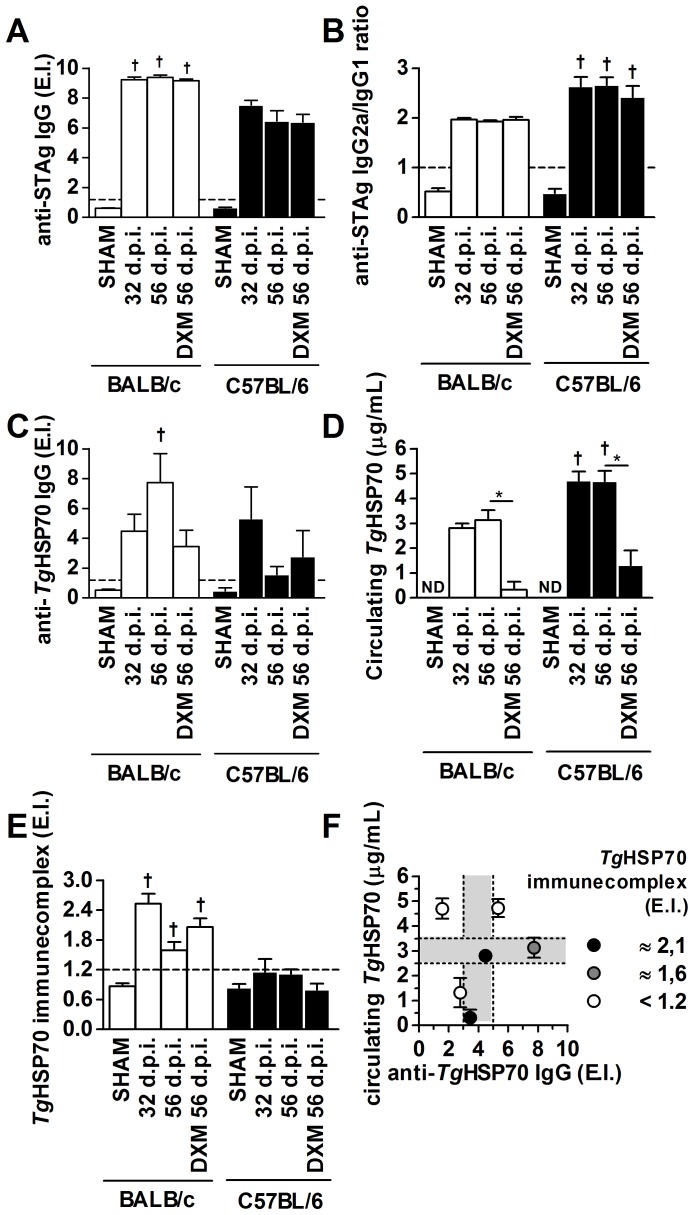
Serological detection of *Tg*HSP70, specific antibodies and immune complexes in *T. gondii* infected animals. Detection of anti-STAg IgG (**A**), anti-STAg IgG2a/IgG1 ratio (**B**), anti-*Tg*HSP70 IgG (**C**), circulating *Tg*HSP70 (**D**) and *Tg*HSP70-specific ICs (**E**) in serum samples of BALB/c and C57BL/6 mice infected with *T. gondii* and/or treated with DXM. Serum samples were collected in different days p.i. as well as from uninfected/untreated mice (SHAM) and analyzed by ELISA. (**F**): Correlation among anti-*Tg*HSP70 IgG (x axis), circulating *Tg*HSP70(y axis) and *Tg*HSP70-IgG IC (dots) obtained from both lineages of mice were analyzed for IC kinetics. Darker dots represent higher amounts of ICs shown in **E**. Optimal conditions (light grey area) for IC formation are represented. ND = non-detected. E.I. = ELISA index (refer to materials and methods section for details). E.I. values above 1.2 (dashed line in **A**, **C**, **D** and **E**) were considered positive. Data are representative of at least two independent experiments of 5 mice per group that provided similar results. *Significant differences between different treatment conditions within the same mouse lineage (ANOVA and Bonferroni multiple comparison post-test; *P<0.05); ^†^Significant differences between the two mouse lineages submitted to the same treatment conditions (Student's *t* test; ^†^ *P*<0.05). d.p.i. = days post-infection.

Both chronically infected mouse lineages (days 32, 56 and DXM-treated mice on day 56 p.i.) presented high STAg-specific antibody titers, with higher titers found in BALB/c than C57BL/6 mice (Figure 3A). In addition, BALB/c presented higher anti-STAG IgG2a (Figure S2 A) and IgG1 (Figure S2 B) antibody titers compared with C57BL/6 mice. However, chronically infected C57BL/6 mice showed higher IgG2a/IgG1 ratio compared with BALB/c mice (Figure 3B). For anti-*Tg*HSP70 antibody detection, additional groups of mice were infected by oral route with 10 *T. gondii* cysts, and it was observed that the seroconversion occurred after 7 days p.i. and in the other time points they present similar specific antibody titers compared with intraperitoneally infected mice (data not shown). Thus, we decided to present the data of *Tg*HSP70 antibody, antigen and immune complexes from day 32 and forth of observation. In this sense, a different response profile from that observed for STAg was seen for *Tg*HSP70. BALB/c and C57BL/6 mice presented anti-*Tg*HSP70 IgG on 32 p.i., and BALB/c mice showed the highest antibody levels on day 56 p.i. However, the DXM-treatment decreased the antibody levels in this mouse lineage (Figure 3C). In contrast, C57BL/6 mice presented decreased anti-*Tg*HSP70 IgG levels on day 56 p.i. as well as when animals were DXM-treated (Figure 3C).

In parallel with the detection of anti-STAg and anti-*Tg*HSP70 IgG we measured the kinetics of circulating *Tg*HSP70 antigen in serum samples at different days of infection and in DXM-treated infected mice (Figure 3D). *Tg*HSP70 was not detected in uninfected mice. BALB/c mice presented circulating *Tg*HSP70 (around 3 µg/mL) at 32 and 56 days p.i., whereas C57BL/6 mice presented higher TgHSP70 concentration (around 4–5 µg/mL) in the same time points. Additionally, DXM-treatment reduced *Tg*HSP70 concentration to 1–2 µg/mL in both infected mouse lineages (Figure 3D). Next, we performed ELISA to detect anti-*Tg*HSP70/*Tg*HSP70 IC in the serum samples of infected animals using *Tg*HSP70-specific IgY as capture antibody. It was observed detectable IC formation in infected BALB/c, but not in C57BL/6 mice. Additionally, the IC detection presented a tendency to be higher on day 32 p.i. (Figure 3E). In accordance with lower and more variable serum antibody detection in C57BL/6 mice, these animals did not present detectable circulating IC. It is known that the formation of IC depends on antigen and antibody concentrations. To better demonstrate the IC formation the data from anti-*Tg*HSP70 IgG (Figure 3C), circulating*Tg*HSP70 (Figure 3D) and circulating IC (Figure 3E) from both lineages of mice were summarized in Figure 3F. It was observed that higher quantities of ICs (darker dots) are present in a region where the *Tg*HSP70 concentration ranges from 2.5 to 3.5 µg/mL and anti-*Tg*HSP70 IgG titers between 3 to 5 ELISA index units (Figure 3F).

### *Tg*HSP70 protein and mRNA expression in the brain were correlated in chronically *T. gondii*-infected mice

**Figure 4:**
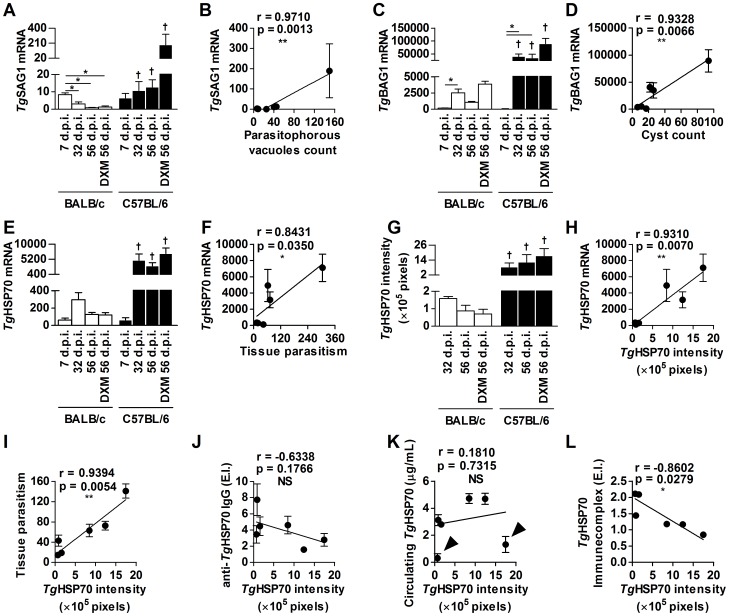
*Tg*HSP70 expression and protein detection in the brain is correlated with parasite replication. Brain samples of BALB/c and C57BL/6 DXM-treated or not and infected with *T. gondii* were processed and analyzed by qPCR for *Tg*SAG1 (**A**), *Tg*BAG1 (**C**) and *Tg*HSP70 (**E**) mRNA expression in the brain. The correlation analysis between parasitophorous vacuoles count in the brain and *Tg*SAG1 (**B**); cyst-like structures and *Tg*BAG1 (**D**) and total parasite count and *Tg*HSP70 (**F**) mRNA expression was performed. The *Tg*HSP70 protein expression was measured by quantifying the intensity of brown staining in brain cysts per tissue section (**G**). The correlation of *Tg*HSP70 protein intensity was done between *Tg*HSP70 mRNA expression (**H**), parasite burden (**I**), serum anti-*Tg*HSP70 IgG (**J**), circulating *Tg*HSP70 (**K**), and *Tg*HSP70-IgG immunecomplexes (**L**). Black arrowheads in **K** indicate DXM-treated groups. *Significant differences between different treatment conditions within the same mouse lineage (ANOVA and Bonferroni multiple comparison post-test; *P<0.05; column graphs). Data are representative of at least two independent experiments of 5 mice per group that provided similar results. ^†^Significant differences between the two mouse lineages submitted to the same treatment conditions (Student's *t* test; ^†^ *P*<0.05;). The correlation coefficient was calculated by the Pearson test (*P<0.05; *P<0.01; *P<0.001; dot plot graphs). d.p.i. = days post-infection.

We performed qPCR for the quantification of total SAG1 and BAG1 mRNA to address tachyzoite and bradyzoite parasitism detection, respectively, in the brain of mice infected and/or treated with DXM. It was observed that on day 7 p.i. BALB/c mice presented the highest SAG1 mRNA expression when compared with chronically infected mice (32, 56, and 56 days p.i. with DXM treatment; *P*<0.05) (Figure 4A). In contrast, C57BL/6 mice on day 7 p.i. did not present significant difference among those infected on days 32 and 56, whereas DXM treatment increased SAG1 mRNA expression in the brain (Figure 4A). These observations were positively correlated with parasitophorous vacuoles count in the brain detected by immunohistochemistry (*r* = 0.9710; p = 0.0013) (Figure 4B and Figure S1 C). Related to BAG1 mRNA, its expression was observed on 32 p.i. and forth in both mouse lineages, being higher in C57BL/6 than in BALB/c chronically-infected mice (Figure 4C), and these levels were correlated to cyst-like structures in the brain (*r* = 0.9328; p = 0.0066) (Figure 4D and Figure S1 D). Additionally, despite not statistically significant, the DXM treatment induced an increase in BAG1 mRNA expression in both mouse lineages (Figure 4C). *Tg*HSP70 mRNA in the brain was also quantified by qPCR. BALB/c mice presented similar *Tg*HSP70 mRNA expression in the brain in acute and chronic phases of infection and also in DXM-treated mice (Figure 4E). *Tg*HSP70 mRNA was detected in the brain of both mouse lineages on day 7 p.i. The *Tg*HSP70 transcripts were higher in the organ of C57BL/6 mice on days 32, 56 p.i. and in DXM-treated mice, and in addition, the expression was higher than that observed in BALB/c mice (*P*<0.05), but no difference was observed between chronically infected C57BL/6 mice treated or not with DXM (Figure 4E). Likewise, *Tg*HSP70 mRNA levels were positively correlated with total tissue parasitism in the brain (*r* = 0.8431; p = 0.035) and indicated that *Tg*HSP70 mRNA expression depends on parasite number in the brain (Figure 4F).

In order to observe the correlation between *Tg*HSP70 protein and mRNA expression in the brain, immunohistochemistry assays were performed to detect *Tg*HSP70 using the specific IgY. Brain tissue slices obtained from all groups were immunoperoxidase stained and different cyst brown-stain patterns were observed (Figure S3 A). Next, the images were acquired and analyzed by ImageJ color deconvolution plugin, which transforms image brown staining into a new grey-scale image (Figure S3 B). Poorly-stained cysts presented mostly light-grey pixels (Figure S3 B, panel a); strongly-stained cysts presented mostly dark-grey pixels (Figure S3 B, panel c); and mild-stained cysts presented grey intermediate staining intensities (Figure S3 B, panel b). Cyst areas were delimited and quantified by ImageJ histogram tool, each cyst presenting unique brown (DAB)-intensity curves. Binary-scale (0 to 255, in which 0 means black and 255 means white) was linearly converted to 0-to-1 scale, in which 0 means white and 1 means black. Therefore, the poorly stained cysts presented a half-Gaussian curve from 0 to 0.2, the mild-stained cysts presented a Gaussian curve from 0 to 0.7 and the strongly-stained cysts presented a Gaussian-like curve between 0.7 to 0.95 (Figure S3 C). *Tg*HSP70 protein quantification was determined by the sum of each brown intensity value multiplied by its respective pixel frequency. Hence, visually different stained cysts presented different *Tg*HSP70 intensity values (Figure S3 D).

Afterwards, this method of *Tg*HSP70 measurement was applied to quantify the protein expression in the brain of *T. gondii*-infected BALB/c and C57BL/6 mice. BALB/c mice infected for 32 days presented higher frequency of mild-stained cysts and this frequency decreased in the brain tissue samples from mice on days 56 p.i. and those DXM-treated mice, respectively (Figure S3 E). In contrast, DXM-treated C57BL/6 mice presented the highest brown-intensity pixels, whereas 32 and 56 days-infected mice presented similar histogram profiles (Figure S3 E). Additionally, brown-intensities above 0.7 were commonly observed in C57BL/6 but not in BALB/c mice (Figure S3 E).

No difference was observed on *Tg*HSP70 detection in the brain among BALB/c mice on days 32, 56 p.i. and in DXM-treated animals (Figure 4G), and these data are in accordance with specific mRNA expression (Figure 4E). Interestingly, the *Tg*HSP70 expression per tissue section of C57BL/6 mice ranged from 8.5×10^5^ to 17.4×10^5^ pixels, which was significantly higher than that observed in BALB/c mice (0.7×10^5^ to 1.6×10^5^ pixels) (Figure 4G). Additionally, the *Tg*HSP70 protein detection was positively correlated with the mRNA expression in the brain in both lineages of mice (*r* = 0.9310; p = 0.0070) (Figure 4H).

*Tg*HSP70 levels in the brain were also associated with total parasite burden (*r* = 0.9394; p = 0.0054) in both mouse lineages (Figure 4I). Nonetheless, *Tg*HSP70 protein levels in the brain were poorly associated with anti-*Tg*HSP70 IgG (Figure 4J) or circulating *Tg*HSP70 (Figure 4K) in both BALB/c and C57BL/6 mice. Interestingly, *Tg*HSP70 levels were inversely correlated with *Tg*HSP70 IC (*r* = −0.8602; p = 0.0279) in BALB/c and C57BL/6 mice in all conditions (Figure 4L).

## Discussion

It was previously shown that *Tg*HSP70 is expressed during *T. gondii* stage conversion from tachyzoite to bradyzoite [2] and transiently from bradyzoite to tachyzoite [3]. Additionally, *Tg*HSP70 is a tachyzoite-specific virulence factor, which increases rapidly just before the death of the host [6] and the specific protein of virulent strains decrease the NF-κB activation and nitric oxide production in host cells [5]. It was also verified that oral *T. gondii* infection with the Fukaya strain induced transient *Tg*HSP70-specific antibodies in BALB/c and C57BL/6 mice, and the higher antibody titers were produced by susceptible C57BL/6 mice [11].

Considering these pieces of information, we were interested in knowing whether the detection of circulating *Tg*HSP70, *Tg*HSP70-specific antibodies and specific ICs could be used for the diagnosis of active toxoplasmosis in reactivation of infection when immunosupression of the host occurs. For this purpose, we first produced the r*Tg*HSP70 protein and then anti-*Tg*HSP70 IgY antibodies. In the present investigation anti-*Tg*HSP70 IgY was obtained from immunized chicken egg yolks by Na_2_SO_4_ precipitation with 90% purity as previously described [26]–[28]. Similarly to hen immunization with STAg [14], anti-*Tg*HSP70 IgY antibodies presented high avidity and were able to detect both recombinant protein and natural protein present in STAg. Thus, the purified *Tg*HSP70-specific IgY antibodies were subsequently used in ELISA and immunohistochemistry as an important immunological tool.

In the next step, susceptible C57BL/6 and resistant BALB/c mice were infected with *T. gondii* and accompanied for the parasite load, histology in the brain and specific seroconversion kinetics. In the chronic phase of infection, the animals were treated with DXM to induce infection reactivation, as previously described [29], [30]. Treatment of mice with DXM is a model that diminishes immune responses and leads to *T. gondii* reactivation, as detected by the increase in the total parasitism and tachyzoite detection, and symptoms of cerebral toxoplasmosis [19], [29], [31]. In accordance with previous studies [29], it was observed a decreasing in cerebral inflammation in chronically infected DXM-treated animals.

Despite reduced inflammatory responses in the brain, DXM-treated C57BL/6 mice presented progressive clinical signs of toxoplasmic encephalitis, such as ruffled coat, tottering walk and locomotion difficulties as previously observed in immunosuppressed and *T. gondii* infected rats [30] and mice [29], [31], [32]. *T. gondii*-infected DXM-treated C57BL/6 mice presented similar symptoms as do untreated mice in the same period of infection. As demonstrated previously C57BL/6 mice presented higher parasite load in the brain than BALB/c mice, on days 32 [33] and 56 p.i., despite not statistically significant. DXM treatment induced an increase in brain tissue parasitism in C57BL/6 mice in parallel with a decreasing in tissue inflammation. In BALB/c mice, the DXM treatment had little immunosuppressive effect, since animals presented similar parasite load as untreated and infected mice in the same period of observation. Similar findings with no significant change in tissue parasitism in the brain of DXM-treated BALB/c mice were observed in previous studies in Wistar rat models of immunosuppression and *T. gondii* infection [30]. However, several studies inducing *T. gondii* reactivation by immunosuppression with DXM have shown contradictory results. The DXM treatment increased the parasitism in the brain of Swiss mice infected with 10 cysts of ME49 strain by oral route [32], increased the p30 positivity in BALB/c mice infected orally with 25 cysts of 76K strain [19] and the tachyzoites in brain, liver, lung, heart and spleen of ICR mice orally infected with 20 cysts of Otoole strain [31]. These data are in accordance with our data of immunosuppressed C57BL/6 mice, which showed an augmented total parasitism and the presence of tachyzoites in the brain. However, the opposite effect was observed for DXM-treated infected BALB/c mice, showing that different mouse lineages, doses of immunosuppressive drug, as well as the *T. gondii* strain used in each experiment, may lead to different results as observed in our experimental design.

For *T. gondii*-specific IgG and IgM detection in serum samples the recognition of recombinant antigens by immunoglobulins present major advantages for the diagnosis of *T. gondii* infection, such as the precise antigen composition of the test is known, more than one defined antigen can be used, and the method can easily be standardized. Additionally, selected antigens could be used to distinguish acute from chronic infections [34]. In the present study, the *Tg*HP70-specific IgG responses differed from those observed for STAg. Whereas anti-STAg IgG titers did not differentiate between chronically infected groups, *Tg*HSP70-specific IgG detection showed different profiles between each group of BALB/c and C57BL/6 mice. The anti-*Tg*HP70 IgG detection reached a peak on day 56 p.i. in BALB/c, and 32 p.i. in C57BL/6 mice, and the titers decreased in this mouse lineage with the progression of infection. Our kinetics data of anti-*Tg*HSP70 IgG detection in serum samples of C57BL/6 mice are in accordance with previous studies infecting mice by oral route with the Fukaya strain [11], however, in contrast to those studies, we observed that BALB/c mice infected with ME49 strain presented higher anti-*Tg*HSP70 levels in serum samples on day 56 p.i. compared with C57BL/6 mice. These different results observed in our study compared with those from [11] could be explained by the different route of infection and strain of the parasite used. Additionally, both mouse lineages respond differently to *T. gondii* infection. *T. gondii* DX strain-infected C57BL/6 mice presented lower levels of antibodies against recombinant GRA1, GRA6, GRA7, p35 and TLA (*Toxoplasma* lysate antigen) than BALB/c mice [35]. In the present study, it was also observed that C57BL/6 presented lower levels of anti-STAg and anti-*Tg*HSP70 IgG than BALB/c mice. Moreover, the DXM treatment was capable to reduce STAg-specific antibody titers and cytokine concentration in the serum of mice infected with the 76K strain [19].- However, in our investigation the DXM treatment did not interfere in anti-STAg antibody titers, but decreased the levels of anti-*Tg*HSP70 antibodies related to the peak of detection in both mouse lineages. This fact may be explained by the differences in experimental procedures, cyst numbers, route of infection and the strain of the parasite used [36]. Clones of Th1 and Th2 cells specifically induce antigen-specific B cells to secrete IgG2a and IgG1, respectively [37]. In our investigation it was shown that despite higher IgG1 and IgG2a antibody levels observed in BALB/c compared with C57BL/6 mice, C57BL/6 presented higher IgG2a/IgG1 anti-STAg ratio according to the higher inflammatory response developed in this mouse lineage under *T. gondii* infection.

In the present study, IgY antibody was used to detect circulating *Tg*HSP70 in the infected mouse sera. Due to their unique properties, chicken immunoglobulin has been used in sandwich ELISA for the detection of circulating antigens [27], [38], [39]. Interestingly, in the present study, higher *Tg*HSP70 concentrations were detected in susceptible, C57BL/6 mice, than in resistant, BALB/c mice, with *T. gondii* infection. A previous report showed that virulent *T. gondii* strains express *Tg*HSP70 in response to immunological stress as a parasite protection mechanism [5], [40], but virulent strains of *T. gondii* (RH, ToxoENT and ToxoP) do not express the HSP70 when hosts are immunosuppressed by cortisone acetate [40]. In our study, DXM treatment reduced significantly the concentration of *Tg*HSP70 in the sera of both mouse lineages, in parallel with lower antibody levels in these animals. Because *Tg*HSP70 is present in the cytoplasm of the parasite [2], [41] and chronically infected mice present the majority of parasites localized into the brain [42], our data suggest that the parasite death by the immune response is the source of *Tg*HSP70 release into the bloodstream. In addition, the higher *Tg*HSP70 detection in the sera of C57BL/6 in comparison with BALB/c mice could be due to the higher parasitism and inflammatory changes observed in susceptible infected mice. As the DXM treatment diminished the inflammatory changes in the brain and probably parasite death, it could be associated with the decrease of the *Tg*HSP70 detection in the sera. In chronically infected C57BL/6 mice treated or not with DXM, the higher *Tg*HSP70 detection in the brain seems to be associated with higher parasite replication compared with BALB/c mice.

In the present investigation, *Tg*HSP70-IgG ICs were detected in the serum samples of BALB/c mice, but not in C57BL/6 mice. In parallel higher levels of antibodies against STAg and *Tg*HSP70 were detected in BALB/c compared with C57BL/6 mice.

IC formation depends on antigen and antibody concentrations. Precipitate formation is higher when proportion of antigen-antibody is within equivalence zone and it is reduced whether antigen and antibody concentration are in excess [43]. The combined detection of soluble SAG-1 and antigen/antibody ICs in the cerebrospinal fluid of children in the presence of clinical findings are consistent with active *Toxoplasma* infection [24]. Also, sera selected from patients with clinical symptoms of toxoplasmosis presented more circulating ICs [44], as well as sera from clinically ill cats and cats with ocular signs of toxoplasmosis [45]. In our study, *Tg*HSP70-specific ICs were only observed in BALB/c mice and they presented lower levels of ICs at 56 days p.i. In the present study, the persistent IC detection in BALB/c infected mice is probably associated with the concentration of *Tg*HSP70 and anti-*Tg*HSP70 in an equivalence zone. ICs were not observed in C57BL/6 mice, indicating that a high *Tg*HSP70 concentration and a low anti-*Tg*HSP70 antibody available in the serum do not favor IC formation in this mouse lineage. Overall, these data indicate that the detection of *Tg*HSP70 antigen, anti-*Tg*HSP70 IgG and *Tg*HSP70-specific ICs in the serum samples can be used as an alternative marker of *T. gondii* infection in resistant BALB/c, but not in susceptible C57BL/6 mice.

Mice infected with *T. gondii* cyst-forming strain present parasites mainly in the brain during chronic phase of infection [42]. Additionally, reactivation of chronic infection by *T. gondii* due to the TNF neutralization [46] or simultaneous depletion of CD4^+^ and CD8^+^ T lymphocytes [47] occurs mainly in the brain with high parasite burden that expresses the tachyzoite marker. In the present study, BALB/c and C57BL/6 mice were infected and in chronic phase of infection they were immunosuppressed with DXM to observe the *Tg*HSP70, SAG1 and BAG1 expression in the brain. Acute infected BALB/c mice (7 days p.i.) presented high levels of SAG1 mRNA, suggesting the presence of tachyzoites. With the progression of infection it was observed a decrease of SAG1 and an increase in BAG1 mRNA expression, and DXM treatment was not able to augment the SAG1 mRNA expression in BALB/c mice. However, in accordance with the increase of tissue parasitism of C57BL/6 mice, it was verified an increase in both SAG1 and BAG1 mRNA expression, and DXM-treatment was able to augment the SAG1 mRNA levels compared with mice of the same lineage on day 56 p.i. In order to reactivate the toxoplasmic infection, DXM treatment is used to induce stage conversion of latent bradyzoites to tachyzoites [19], [31], [48], which changes the antigen profile, diminishing BAG1 and increasing SAG1 expression in the brain [31]. Thus, with our experimental protocol the DXM treatment was able to induce stage conversion of *T. gondii* in the brain of C57BL/6, but not in BALB/c mice. Glucocorticoids are important modulators of immune cell functions (reviewed by [49]) and are widely used in clinical practice, being effective in the treatment of autoimmune disease [50], immunological rejection [51], and atopic dermatitis [52]. In accordance, toxoplasmosis was observed in patients suffering bone marrow transplantation (BMT); from 9 cases of disseminated *T. gondii* infection in BMT transplantation, six patients received corticosteroids treatment in addition to others immunosuppressive drugs and the main affected organ was the brain [53]. Thus, glucocorticoids treatment can contribute to active toxoplasmosis in human patients.

It is stated that, under stress conditions, *T. gondii* expresses *Tg*HSP70 during tachyzoites and bradyzoites stage conversion and *vice versa* [2], [3]. In our present investigation, infected and DXM-treated or not C57BL/6 mice showed high levels of *Tg*HSP70 mRNA in the brain, demonstrating that the parasite stage differentiation occurs normally in this lineage of chronically infected mice. Also, *Tg*HSP70 mRNA expression was highly correlated with *Tg*HSP70 protein quantification in the brain sections. Accordingly, by photometric assay, C57BL/6 mice treated with anti-IFN-γ and anti-TNF antibodies resulted in homogeneous increase in *Tg*HSP70 expression inside brain tissue cysts [3].

Heat shock proteins are highly conserved chaperones involved in several processes inside cells for maintaining cell homeostasis under stress conditions, processing antigens to presentation, regulating apoptosis, among others [54]. However, HSP70 release develops important roles for Apicomplexan protozoan parasites as a mechanism of modulating host immune responses [5], [7], [55]. The data obtained here demonstrated that chronically infected C57BL/6 mice presented high tachyzoite multiplication rate that became higher under immunosuppression, and in this period of the life cycle of the parasite the *Tg*HSP70 expression occurred. Thus, in C57BL/6 mice the *Tg*HSP70 is better related to the parasite replication than stress of the organism, because the DXM treatment diminished the tissue pathology.

Taken together, the data obtained in the present investigation demonstrate that in C57BL/6 mice, at least in the brain, the *Tg*HSP70 expression by the parasite is associated with high parasite replication, indicating active toxoplasmosis and/or reactivation of infection. However, the *Tg*HSP70 release into the bloodstream is associated with the immune response, since DXM-treated mice presented lower antigen detection in serum samples. Additionally, the detection of anti-*Tg*HSP70 IgG antibodies in parallel with specific ICs in the serum is correlated with resistance of the mice.

## Supporting Information

Figure S1 **C57BL/6 mice present higher cyst and parasitophorous vacuoles in the brain.** Photomicrograph of typical cyst-like structure in the brain of C57BL/6 mice on day 56 p.i. (**A**) and a parasitophorous vacuole in the lung of C57BL/6 mice on day 7 p.i. (**B**). The quantification of *T. gondii* parasitophorous vacuoles (**C**) and cyst-like structures (**D**) in the brain of chronically infected mice were done by immunohistochemistry assays. Bar scale, 100 µm. Data are representative of at least two independent experiments of 5 mice per group that provided similar results. *Significant differences between different treatment conditions within the same mouse lineage (one-way ANOVA and Bonferroni multiple comparison post-test; *P<0.05). ^†^Significant differences between the two mouse lineages submitted to the same treatment conditions (Student's *t* test; ^†^ *P*<0.05).
(TIF)

Figure S2 **Detection of anti-STAg IgG2a and IgG1 antibodies in the sera of infected mice.** Detection of anti-STAg IgG2a (**A**) and anti-STAg IgG1 (**B**) in serum samples of BALB/c and C57BL/6 mice treated or not with DXM and infected with *T. gondii*. Serum samples were collected in different days p.i. as well as from uninfected/untreated mice (SHAM) and analyzed by ELISA. E.I. = ELISA index (refer to materials and methods section for details). E.I. values above 1.2 (dashed line in **A** and **B**) were considered positive. Data are representative of at least two independent experiments of 5 mice per group that provided similar results; ^†^Significant differences between the two mouse lineages submitted to the same treatment conditions (Student's *t* test; ^†^ *P*<0.05). d.p.i. = days post-infection.
(TIF)

Figure S3 ***Tg*** **HSP70 protein quantification in the brain.** **A**: Brain tissue was stained for *Tg*HSP70 by immunohistochemistry using *Tg*HSP70-specific IgY antibody. Panels indicate photomicrographs of a mild-stained (**a**), intermediate-stained (**b**), and strongly-marked (**c**) cyst-like structure. The images of tissue cyst-like structures, as in A, were obtained and analyzed with ImageJ software using color deconvolution plugin. The pixel DAB-intensity from panels **a**-**c** shown in **B** were quantified by ImageJ software histogram tool (**C**). The sum of the multiplication of each brown-intensity value by its respective pixel frequency was used for determination of *Tg*HSP70 intensity of each histogram (**a**–**c**) shown in **C** (**D**). *Tg*HSP70 protein expression in the brain was investigated individually for each group of BALB/c and C57BL/6 (**E**) mice. Threshold line (0.7) indicates brown-intensities present only in strongly-marked cysts. d.p.i. = days post-infection.
(TIF)
