# Supplementary figures and images for: Toxoplasma gondii 70 kDa Heat Shock Protein: Systemic Detection Is Associated with the Death of the Parasites by the Immune Response and Its Increased Expression in the Brain Is Associated with Parasite Replication
Source: PLoS One. 2014 May 6;9(5):e96527. doi: 10.1371/journal.pone.0096527 (PMC4011789; doi:10.1371/journal.pone.0096527)

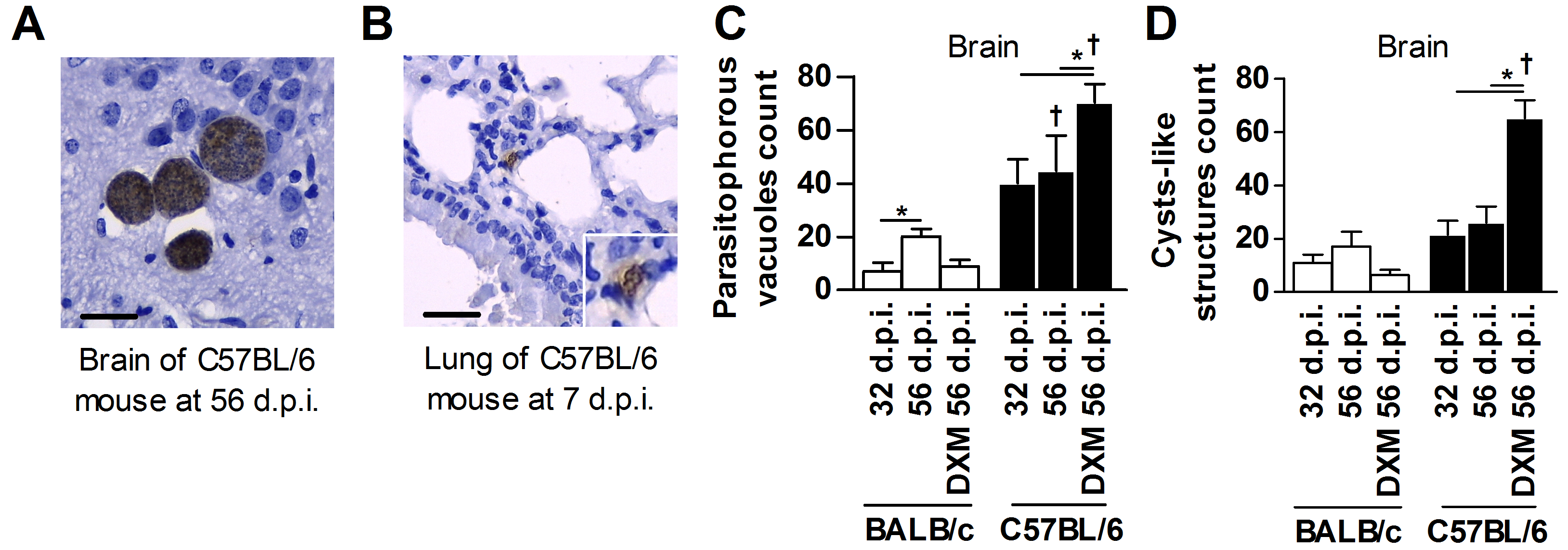

Supplement: Figure S1 — C57BL/6 mice present higher cyst and parasitophorous vacuoles in the brain. Photomicrograph of typical cyst-like structure in the brain of C57BL/6 mice on day 56 p.i. (A) and a parasitophorous vacuole in the lung of C57BL/6 mice on day 7 p.i. (B). The quantification of T. gondii parasitophorous vacuoles (C) and cyst-like structures (D) in the brain of chronically infected mice were done by immunohistochemistry assays. Bar scale, 100 µm. Data are representative of at least two independent experiments of 5 mice per group that provided similar results. *Significant differences between different treatment conditions within the same mouse lineage (one-way ANOVA and Bonferroni multiple comparison post-test; *P<0.05). †Significant differences between the two mouse lineages submitted to the same treatment conditions (Student's t test; † P<0.05). (TIF) [file pone.0096527.s001.tif]

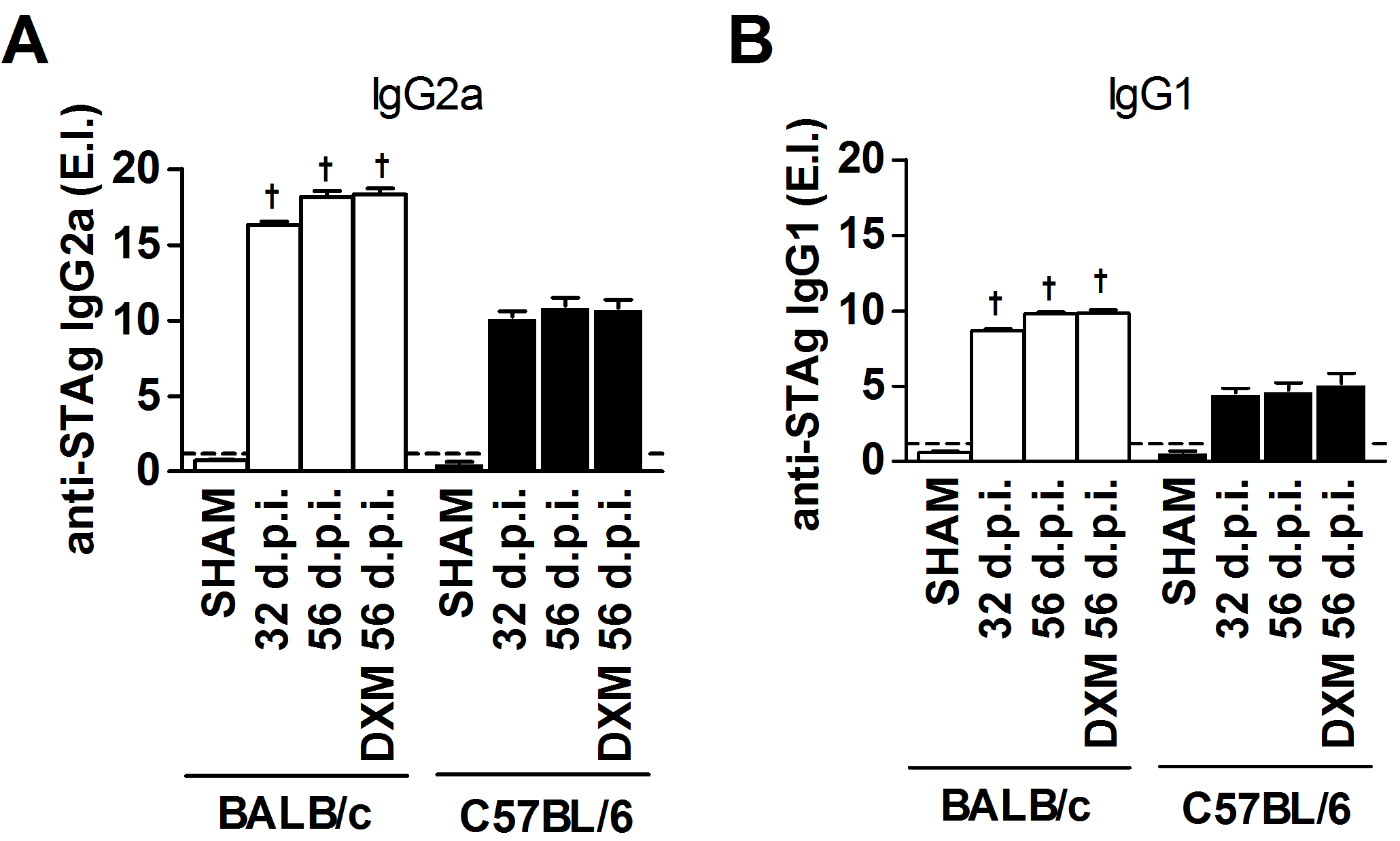

Supplement: Figure S2 — Detection of anti-STAg IgG2a and IgG1 antibodies in the sera of infected mice. Detection of anti-STAg IgG2a (A) and anti-STAg IgG1 (B) in serum samples of BALB/c and C57BL/6 mice treated or not with DXM and infected with T. gondii. Serum samples were collected in different days p.i. as well as from uninfected/untreated mice (SHAM) and analyzed by ELISA. E.I. = ELISA index (refer to materials and methods section for details). E.I. values above 1.2 (dashed line in A and B) were considered positive. Data are representative of at least two independent experiments of 5 mice per group that provided similar results; †Significant differences between the two mouse lineages submitted to the same treatment conditions (Student's t test; † P<0.05). d.p.i. = days post-infection. (TIF) [file pone.0096527.s002.tif]

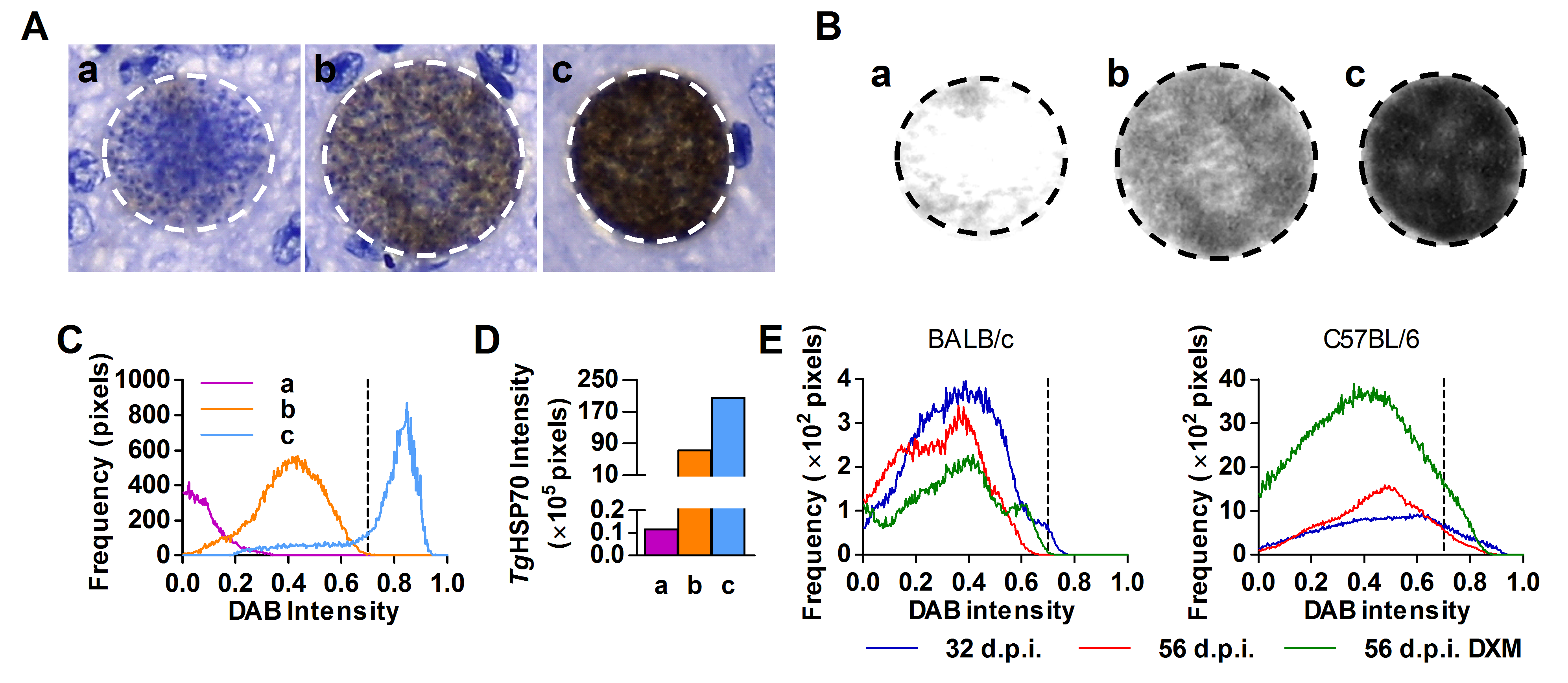

Supplement: Figure S3 — Tg HSP70 protein quantification in the brain. A: Brain tissue was stained for TgHSP70 by immunohistochemistry using TgHSP70-specific IgY antibody. Panels indicate photomicrographs of a mild-stained (a), intermediate-stained (b), and strongly-marked (c) cyst-like structure. The images of tissue cyst-like structures, as in A, were obtained and analyzed with ImageJ software using color deconvolution plugin. The pixel DAB-intensity from panels a-c shown in B were quantified by ImageJ software histogram tool (C). The sum of the multiplication of each brown-intensity value by its respective pixel frequency was used for determination of TgHSP70 intensity of each histogram (a–c) shown in C (D). TgHSP70 protein expression in the brain was investigated individually for each group of BALB/c and C57BL/6 (E) mice. Threshold line (0.7) indicates brown-intensities present only in strongly-marked cysts. d.p.i. = days post-infection. (TIF) [file pone.0096527.s003.tif]
